# Enhancing primary care quality improvement through national data collection and validation: the primary care quality initiative in Sweden

**DOI:** 10.1080/02813432.2025.2490921

**Published:** 2025-04-20

**Authors:** Jörgen Månsson, Malin André, Emil Johansson, Charlotta Malmer Hagstam, Maria C. M. Eriksson, Susanne Steen, Ulrika Elmroth, Eva Arvidsson

**Affiliations:** a General Practice/Family Medicine, School of Public Health and Community Medicine, Institute of Medicine, Sahlgrenska Academy, University of Gothenburg, Gothenburg, Sweden; b Department of Public Health and Caring Sciences, Family Medicine and Preventive Medicine, Uppsala University, Uppsala, Sweden; c Department of Data and Analysis, Group staff Digitization, Group Office, Västra Götaland Region, Sweden; d Department of Clinical Sciences, Lund University, Malmö, Sweden; e University Clinic Primary Care Skåne, Region Skåne, Sweden; f Research, Education, Development & Innovation, Primary Health Care, Region Västra Götaland, Sweden; g Department of Care and Welfare, the Swedish Association of Local Authorities and Regions, Sweden; h Department of Health, Medicine and Caring Sciences, Linköping University, Linköping, Sweden

**Keywords:** Primary care, quality improvement, quality indicators, data collection, validation, healthcare analytics, electronic health record

## Abstract

**Objective:**

Quality measures in healthcare are crucial for improving outcomes and ensuring patient safety. This study investigated the evolution, implementation, and impact of Primary Care Quality (PCQ). The PCQ aims to facilitate nationwide quality benchmarking, serving as a tool for quality improvement (QI) and research.

**Design/Settings:**

A descriptive design outlining the development and operationalisation of the PCQ, a national framework for automatic and systematic data collection and feedback.

**Results:**

The national PCQ system is a tool for continuous QI in primary care in Sweden. PCQ has achieved extensive adoption, with over 97% of Swedish primary care centres, both private and public driven, utilising the platform for automatic data extraction from patient records and data visualisation. Quality indicators were developed through a structured approach involving primary care professionals, evidence-based clinical practices, and expert contributions from established knowledge organisations, reflecting the breadth of general practice. Data are automatically retrieved from medical records and visualised in real time, with the possibility of benchmarking at an aggregate level and identifying individuals locally at primary care centres. The PCQ has facilitated improvements by enabling quality dialogue among healthcare professionals and supporting continuous local QI. Regionally, the PCQ supports needs assessments and patient safety initiatives. Nationally, it establishes standardised indicators for quality measurement, enabling effective benchmarking and strategic healthcare planning.

**Conclusions:**

The implementation of the national PCQ system provided a framework and tool for continuous QI in primary care. The system has influenced national standardization of primary care indicators, with quality improvement results demonstrated regionally and locally through the PCQ.

## KEY POINTS

Primary Care Quality is a national system designed for automatic data collection and feedback from primary care electronic medical records.

- Primary Care Quality aims for quality improvement, consists of indicators developed by professionals, and reflects the breadth of general practice.
- Data is visualised in real time as graphs with the possibility of benchmarking
- Primary Care Quality is available for over 97% of all primary care centres in Sweden.

## Introduction

Over the past decade, there has been a growing emphasis on enhancing measures for quality improvement (QI) in healthcare systems worldwide [1]. In Sweden, the ‘PrimärvårdsKvalitet’, the Primary Care Quality (PCQ) initiative represents a significant advancement in systematically monitoring and improving primary healthcare quality using standardised indicators automatically retrieved from electronic medical records (EMRs). This study delves into the background, methodology, and objectives of the PCQ system, highlighting its role in promoting evidence-based practices and continuous QI and providing possibilities for new research in primary care.

### Quality measurement in healthcare

The field of quality measurement in healthcare has grown substantially, with increasing global interest from researchers, policymakers, healthcare providers, and professionals [1–3]. Many countries have developed databases using automated data extraction systems for surveillance, research, and practice improvement [4,5]. Moreover, a few countries provide data feedback to primary care centres (PCCs) for QI [6–9]. In Sweden, initiatives such as the investigation ‘Guldgruvan i hälso- och sjukvården’ (‘The gold mine in healthcare’) in 2010 underscored the importance of leveraging health data, particularly from primary care, to enhance quality registries, inspire QI, and support research endeavours [10]. Many disease-specific quality registers have been developed in Sweden over the years [11]. However, these are not applicable in primary care, where the scope encompasses a wide range of health issues, from benign to malignant conditions, general symptoms, and specific diseases of both acute and chronic nature, often combined with comorbidity. Moreover, manual registration, which is often required in these registers, is not feasible in primary care due to the time required. When the quality of care is scrutinised by the authorities responsible for providing and financing primary care in Sweden, production-related measures such as the number of visits, expenses for drug prescriptions, laboratory tests, and radiographs are often used, while data on clinical measures have been sparse [12].

Primary care in Sweden is defined in the Swedish Health and Social Services Act as outpatient care without limitations in terms of illness, age, or patient groups and is responsible for medical assessment and treatment, nursing, prevention, and rehabilitation that does not require specialised medical or technical resources or skills [13]. The Swedish healthcare system is decentralised and managed at the national, regional, and local (municipal) levels. The 21 regions independently regulate the requirements, funding, and establishment of PCCs. Furthermore, each Swedish individual has a unique personal ID number. All the inhabitants are listed in a PCC, and each PCC is responsible for its listed patients. Most PCCs in Sweden are team-based, with registered nurses, district nurses, general practitioners (GPs), GP trainees, psychologists, psychotherapists, physiotherapists, and occupational therapists working together. In certain regions, physiotherapists and occupational therapists work in separate rehabilitation units instead of PCCs.

The aim of this study is to describe the methods and results of the development and use of PCQ as an emerging system, its quality indicators, and PCQ as a tool for QI.

## Primary care quality as a system

The development of the PCQ system in Sweden reflects the long-standing need for metrics to support PCCs with quality data in their improvement efforts and evaluate healthcare outcomes in primary care. A national system for automatic collection and feedback of primary care data can be traced back to the early 2000s when most PCCs in Sweden adopted electronic medical records (EMR) but lacked a unified system for extracting and aggregating data at local, regional, or national levels. The National Board of Health and Welfare had initiated studies to collect patient data from EMRs [14,15]. However, implementation and further development were postponed as each region developed its own regional databases and tools for QI. In 2011, three different primary care initiatives sought funding from the Organization for National Quality Registries, leading to a joint venture and eventually the establishment of the PCQ.

A working group comprising representatives from the three projects, including GPs and district nurses, initiated the development of the PCQ. Over time, the group navigated various funding sources, such as research grants, quality registry funds, and financing from the National Board of Health and Welfare. Legal and ethical concerns had led to a shift by 2014 from creating a centralised database with individual patient data to managing individual data at the PCC level, with only aggregated data collected and presented regionally and nationally. The Swedish Data Protection Authority (IMY) observed that the patient registry already involves extensive collection and processing of sensitive personal data without the patient’s consent. A proposed expansion of the patient registry would result in a significantly increased processing of highly sensitive personal data concerning large portions of Sweden’s population.

The aim was to support QI through analysis and reflection based on data within local primary care units, as well as regionally and nationally. In 2015, the project was formally led and financed by the Swedish Association of Local Authorities and Regions (SALAR; Sveriges Kommuner och Regioner, i.e. SKR).

**Figure 1.**
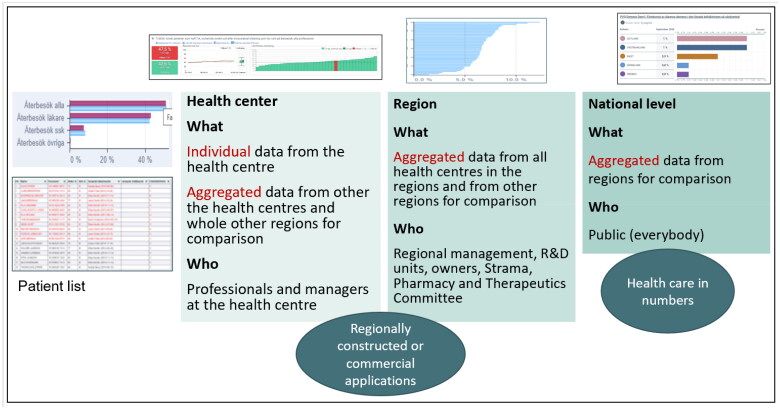
Data presentation at different levels, what is visualised, and who has access.

As a national system, PCQ provides comprehensive access to data reflecting different aspects of quality for PCCs, as well as for regional and national authorities, enabling continuous monitoring and enhancement of clinical practices [16]. Currently, over 97% of PCCs (both public and private driven) and most of the rehabilitation units, representing all 21 regions, participate in PCQ, with data on most PCQ indicators. Data updated monthly from EMRs allow for almost real-time visualisation and analysis of PCQ indicators at the regional and local levels. At the PCCs and rehabilitation units, relevant staff groups can monitor data on outcomes for individual patients and benchmarks against other PCCs and rehabilitation units. However, only aggregated data are available at the regional and national levels (Figure 1). Data withdrawal varies between regions whether if it is possible to access data only from their own primary care center, only public primary care centers, all primary care centers or the entire healthcare system.

Applications for data collection and visualisation at the PCC and rehabilitation units and the regional level differ across regions. Uppsala, Östergötland, and Halland had their applications, whereas the remaining 18 regions used solutions from an independent provider (Medrave Software AB, Stockholm, Sweden).

Nationally, aggregated data for selected indicators are displayed on the common platform ‘Health care in numbers’ (Vården i siffror) [17]. The PCQ working group determines which indicators are suitable for public displays.

Moreover, a new national database for PCQ data, aggregating data from all 21 regions, is currently underway in collaboration with SALAR. Patient privacy was safeguarded in the national database by aggregating data and masking small values to minimise identification risks while preserving data integrity.

Data from the national PCQ database are released to different stakeholders after obtaining approval from the PCQ working group. For example, approved ethical approval is required. In the event of a request from individuals, media, or private companies regarding data from the national database, the request should be redirected to the relevant regions.

The working group for the PCQ now represents a broader range of healthcare professionals, including GPs, nurses, district nurses, psychologists, physiotherapists, and occupational therapists. The group collaborates with national entities, such as the National System for Knowledge-Driven Management, the Public Health Agency, a few disease-specific quality registries, and the National Board of Health and Welfare, to align indicators with the care guidelines.

### Data validation and quality assurance

The validation processes conducted at the regional level were continuously improved to enhance data accuracy and completeness. A network of regional representatives regularly meets to support ongoing validation efforts. Comprehensive validation included control of participating PCCs and rehabilitation units, indicator completeness, and longitudinal trend analysis, with scientific validation ongoing in selected areas. However, at the national level, extraction processes vary between regions due to variability in organisational structures, including reimbursement programs and care practices [18].

## Indicators in PCQ

The indicators were initially planned based on the most common diagnoses in primary care. However, early project financiers stated that quality indicators should be based on evidence-based measures to implement new knowledge from national guidelines and healthcare programs. Furthermore, evaluating the appropriate interventions is difficult for many patient groups, which are common in primary care, such as those with unclear symptoms. Thus, the starting points became chronic diseases, infections, antibiotic treatment, and broad non-disease-specific areas such as continuity and comorbidity. Primary care experts were engaged early to review the indicators based on current treatment recommendations and guidelines.

As the PCQ indicator data are automatically retrieved from existing documentation, the indicators must be based on structured information from EMRs. Therefore, the data consists of patient identity (including sex and age), type of contacts (visit, telephone, video), date for contact, diagnosis codes (ICD 10, KSH97-P), procedure codes (KVÅ), laboratory test results, and drug prescriptions. For each disease, a number of common ICD-10 diagnoses were collected and defined as a group, e.g. diabetes type 2 with and without complications.

The indicators are usually expressed as the percentage of listed patients at the PCC who have a certain disease or have received a certain treatment. Most indicators consist of a numerator and denominator, except for continuity indicators. Health professionals at PCCs can view the results of each indicator over time and benchmark them against other PCCs in the region. Furthermore, the results can be represented separately according to sex and age group. At the PCCs, the data on the individual patients included or not included in a certain indicator can be accessed, for example, which patients with a certain disease had a certain treatment and those who had not.

The project engaged a broad range of primary care professionals during the development process to make the indicators practical for clinical use. New indicators were created based on ideas from professionals at the PCCs, suggestions from guideline producers, and the PCQ working group itself. Interested healthcare professionals were also invited to meetings several times to discuss suggestions and ideas.

Accuracy and validity were tested before implementation. The working group collaborated with other initiatives, such as the National Board of Health and Welfare’s special patient safety initiative, Strama (strategic work against antibiotic resistance), and the National System for Knowledge-Driven Management within Swedish Healthcare [19].

As the construction of indicators in the PCQ developed, certain principles that should be fulfilled emerged (Table 1).

**Table 1.** Principles that should be fulfilled for new indicators in PCQ.

The indicators should
be useful for quality improvements for the individual patient and at the system level be based on evidence from primary care contribute to quality for the patients in primary care contribute to a fair resource allocation among the patients in primary care be functional and reasonable based on the working methods and conditions of primary care be practically and technically possible to produce through automatic data extraction

Approximately 150 quality indicators covering major diseases, public health issues, infections, and overarching aspects, such as continuity, prioritisation, lifestyle habits, nursing, rehabilitation, and comorbidity, exist (see Supplementary Appendix A). Indicators are updated regularly based on current sources of knowledge, such as revised medical guidelines and new medicines. All indicators are defined in ‘Kvalitetsindikatorkatalog – KIK’ [20].

### General indicators for primary care

Continuity, comorbidity, prioritisation of patients with high needs, frequent attendance (calculated from 3% of the most frequent attenders), lifestyle habits, and older adult patients are examples of the indicator areas in this group (Supplementary Appendix A). The continuity indicators were calculated using an index [21], whereas the others were constructed using numerators and denominators.

### Chronic diseases

Indicators of chronic diseases reflect diagnoses such as diabetes, heart failure, chronic obstructive pulmonary disease (COPD), hypertension, and osteoporosis, which are predominantly managed in primary care (Supplementary Appendix A). One indicator for each diagnosis displays the proportion of diagnosed patients and whether they had regular follow-up visits. In many indicators, patients are defined as having a *new* diagnosis if no similar diagnosis occurred in the last few years, as certain interventions are only performed once. Other disease groups are limited to patients using medication regularly, focusing on those with more severe diseases.

### Mental health

The mental health indicators include depression, anxiety, and stress-related diagnoses (Supplementary Appendix A). Similar to chronic diseases, one indicator measures the proportion of patients with a diagnosis, while others assess the proportion undergoing certain evidence-based interventions, such as different types of psychotherapy.

### Nursing

Examples of nursing diagnoses (included in the chapter on chronic diseases) include leg ulcers and urine incontinence. The measurements include the proportion of patients with a certain diagnosis, as well as the proportion of patients who has certain evidence-based interventions. One example is the proportion of patients with leg ulcers and etiological diagnosis.

### Rehabilitation

Most rehabilitation indicators are included in the chapter on chronic disease and measure the proportion of patients with a certain disease who underwent evidence-based rehabilitation interventions, such as the proportion of patients with a certain diagnosis who underwent training supervised by a physiotherapist. New indicators of the proportion of patients with different musculoskeletal diagnoses are currently under development.

### Infections

These indicators concern the most common infections in primary care (Supplementary Appendix A). Several indicators have specified targets for an antibiotic prescription. The indicators of infectious diseases have a slightly different history than other indicators. In Sweden, Strama is a national healthcare professional network promoting responsible antibiotic use [22]. The core activities are outreach visits and discussions on guidelines and antibiotic prescriptions. When the PCQ started, Strama already had worked together with the Public Health Agency of Sweden for a decade to develop indicators of antibiotic prescriptions. Thus, indicators for respiratory tract infections were developed, and additional indicators have been added since then.

### Patient safety

Patient safety indicators in primary care were developed mainly using existing indicators together with National Board of Health and Welfare’s 2019–2020 initiative.

### Sustainable health care

Many indicators from different areas concerning the sustainable use of drugs are gathered in a group, for example, the indicator of the prescription of addictive drugs, antibiotics, and proton pump inhibitors without a relevant diagnosis. Indicators showing the proportion of patients with a certain diagnosis are useful in balancing over- and underdiagnosis when compared with other PCCs.

## PCQ as a tool for quality improvement

The PCQ supports healthcare professionals in identifying areas that may require improvement and enables the implementation of targeted interventions that improve patient outcomes and care standards. Over the years, the PCQ project has actively supported and encouraged the use of PCQ as a QI tool. Initiatives have included annual webinars featuring primary care representatives showcasing QI projects using PCQ data, as well as workshops and seminars conducted by PCQ working groups at professional association meetings. A national superuser network is being established to facilitate the use of PCQ.

**Figure 2.**
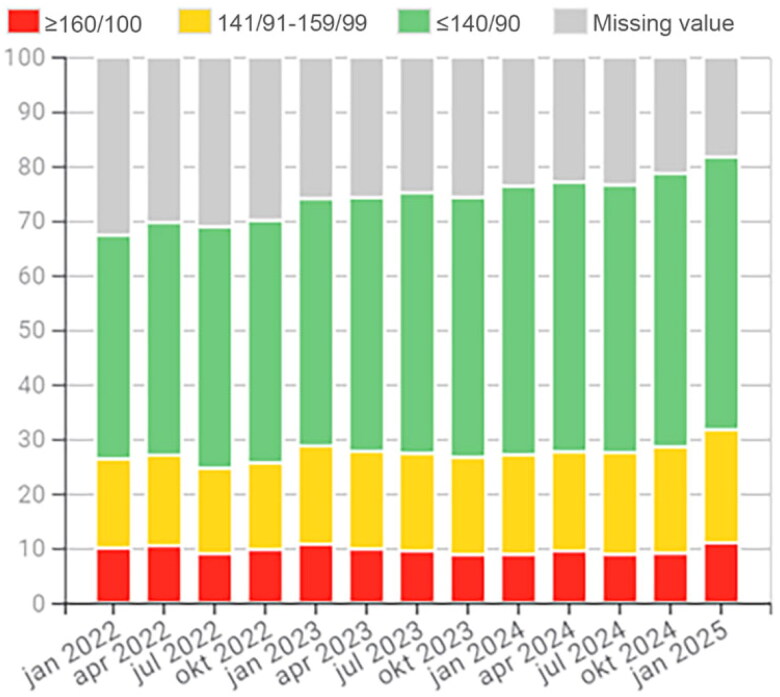
Percentage of patients with hypertension and with a recorded blood pressure of ≥ 160/100 mmHg (red), with a recorded blood pressure of 141/91–159/99 (yellow) and with a recorded blood pressure of ≤140/90 mmHg (green) 2022-01 to 2025-01 for the PCC centrumkliniken.

A nationwide course for GP trainees further promoted PCQ use, yielding positive participant engagement and application outcomes at their PCCs. The PCQ working group has developed extensive online resources to support indicator usage and initiate QI projects, providing practical examples during webinars, such as tracking follow-ups for patients with chronic diseases or identifying missing key measures such as HbA1c in diabetes care or target values for blood pressure (Figure 2).

PCQ indicators have been integrated into structured peer reviews, such as the Collegial Quality Dialogue by the Swedish Society of Medicine [23].

The PCQ collaboration with the Public Health Agency and Strama led to an action plan for promoting proper antibiotic use through PCQ. In the project ‘AntibioticSmart PCC’, PCQ data served as criteria for accreditation [24]. As part of this plan, the National Strama Group arranges several seminars annually to help Strama workers use the indicators.

**Figure 3.**
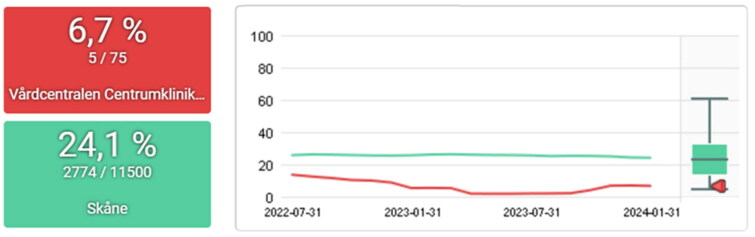
Percentage of episodes with acute bronchitis prescribed antibiotic 2022-09-30 to 2024-03-31 for the PCC centrumkliniken (red) and the region of skåne (green). The target level set by strama is ≤10%.

**Figure 4.**
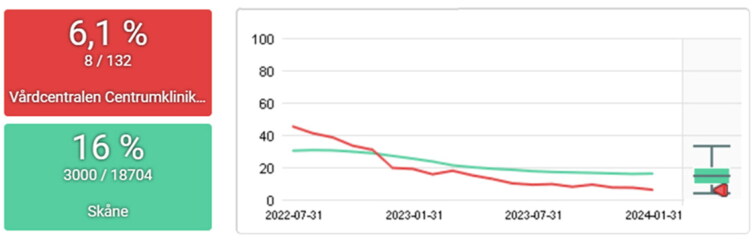
Percentage of episodes with acute pharyngotonsillitis with negative rapid antigen detection tests for group a streptococci prescribed antibiotic 2022-04-30 to 2023-10-31 for the PCC centrumkliniken (red) and the region of skåne (green). The target level set by strama is ≤10%.

**Figure 5.**
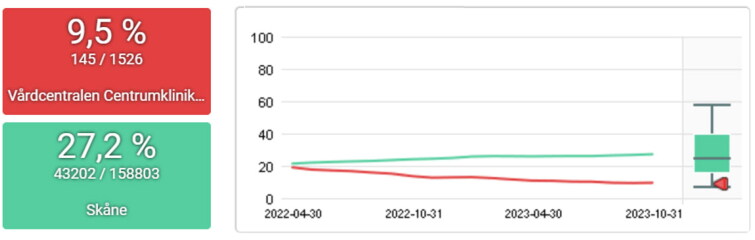
Percentage of tests for C-reactive protein in episodes of an upper respiratory tract infection (common cold, acute pharyngotonsillitis, acute otitis media, and acute sinusitis) 2022-07-31 to 2024-01-31 for the PCC centrumkliniken (red) and the region of skåne (green). The target level set by strama is ≤10%.

**Figure 6.**
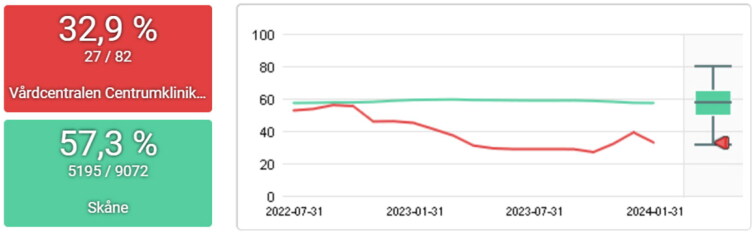
Percentage of episodes with acute sinusitis prescribed antibiotic 2022-07-31 to 2024-01-31 for the PCC centrumkliniken (red) and the region of skåne (green).

A few examples of the progression of PCQ indicators from a PCC working to become accredited as an ‘AntibioticSmart PCC’ are provided in Figures 3–6. In this PCC, data from the PCQ have recurrently been the starting point for dialogue and discussion connected to the national guidelines and targets among colleagues.

Many regions use PCQ indicators for follow-up by funding authorities [25,26], and a few monitor the implementation of the national system for knowledge-driven management. PCQ is used for healthcare planning, for example, to determine the number of patients with specific conditions such as heart failure or COPD across a region, aiding in the prioritisation of interventions that address the most pressing healthcare needs. PCQ indicators developed for monitoring and improving patient safety have been used to enhance patient safety in certain regions. In certain regions, PCQ indicators have been used in structured peer reviews [23].

## Discussion

The national PCQ system has become a key tool for continuous QI in Swedish primary care settings. The PCQ is designed for local QI, consists of indicators developed by professionals, and reflects the breadth of general practice. Data were automatically retrieved from the EMRs and visualised in real time as graphs, allowing benchmarking at an aggregated level and identifying individual patients locally at the PCC.

The work with the PCQ has had a national impact in creating standardized indicators for primary care, including important measures such as continuity of care and comorbidity. At the regional level, results can be seen in the reduction of antibiotic prescriptions through the Strama initiative, which utilizes the PCQ system. Other notable results could be seen regionally on other indicators (16). Additionally, the outcomes of local QI efforts are demonstrated in the results section (Figures 2–6).

### PCQ as a tool for quality improvement

Several international studies have concluded that using data retrieved from EMRs in QI can improve both efficiency and clinical practice [3,27–31]. In these studies, data were represented as dashboards that provided immediate access, complemented by benchmarking. The integration of quality indicators and benchmarking has proven valuable in Sweden. A correlation between the use of the PCQ and improved healthcare quality has recently been demonstrated [16].

Results further improve when feedback is combined with other interventions such as outreach visits, participants’ meetings, or QI plans [32,33]. For example, Sweden’s long-standing initiative to reduce antibiotic prescriptions, which incorporates PCQ data alongside outreach visits, has contributed to measurable reductions in antibiotic use [34–36].

The tradition of quality circles, which are common in primary care in many European countries, is less widespread in Sweden [37–39]. Often, the starting point for quality circles and group discussions is patients or data derived from the participant’s own practices, but a common challenge has been the lack of current data to support these discussions [38]. The PCQ facilitates this process by providing relevant data on various health issues. Supplementing the allocated time for group discussions and quality circles with this data is important [12].

Quantitative measures provide valuable insights, but they cannot fully capture the complexities of health care quality. While factors such as patient satisfaction surveys, waiting times, and clinical outcomes can be assessed using quantitative methods, other elements are more difficult to quantify. These include aspects like the quality of the patient-provider relationship, cultural competence, patient-centered care and the overall experience of care.

### Indicators in PCQ

The PCQ system in Sweden is a comprehensive framework of quality indicators grounded in evidence-based guidelines developed by primary care professionals in collaboration with experts from academic institutions, the National Board of Health and Welfare, the Public Health Agency, and other organisations, resembling the approach described by other authors [40]. The PCQ is integrated within Sweden’s broader knowledge organisation, which includes collaboration with specific quality registries and primary care professional associations. However, to balance requests from other stakeholders to measure different aspects of primary care and create indicators that are useful in daily practice, certain principles for new indicators have been adopted. Similar principles have been described previously [1,32,33]. Indicators in the PCQ are considered relevant if they are consistent with established goals and priorities, recommend actions that can be improved, fall under the recipient’s control, and are comparable with those of other health providers, where possible. These indicators do not require additional documentation [41].

As part of designing PCQ indicators that are relevant to primary care and consistent with everyday clinical activities, the indicators reflect aspects such as continuity and multimorbidity, addressing the nuanced needs of general practice. The challenges in constructing quality indicators that adequately reflect the patient-centred nature of primary care, rather than a disease-centred approach, have been addressed in earlier studies [2,42]. Most indicators associated with chronic diseases are based on national guidelines. However, many patients have several diseases and may gain more from person-centred adaptation of the guidelines than from strict adherence [43]. Therefore, indicators measuring comorbidity and inappropriate drug treatments have been developed to provide a more balanced picture of clinical quality. The PCQ contains several indicators of nursing, psychotherapy, and rehabilitation interventions, reflecting a multiprofessional approach. The inclusion of a broader range of primary care diagnoses, including unspecified symptoms—which was the initial goal of the PCQ—has proven difficult and has not yet commenced. Furthermore, the PCQ system does not include patient-related outcome measures or aspects of patient experiences [44,45].

### PCQ as a system

The major advantage of a PCQ system is its extensive coverage. With over 97% of Swedish PCCs participating, the PCQ indicators are now the national standard for quality indicators in primary care.

The PCQ provides PCCs with real-time data, facilitating QI efforts while reducing manual reporting requirements by automatically extracting data from EMRs [1,35,40]. The coding of diagnoses and procedures is a crucial step in retrieving data for indicators. Swedish GPs are accustomed to diagnostic coding because ICD-10 codes are used as part of the reimbursement system for PCCs. However, they are less accustomed to use procedure code, e.g. using codes for different types of treatment. Other professionals are generally more accustomed to procedural coding and less familiar with diagnostic coding, although they can diagnose diseases or symptoms according to their competence.

Automated data extraction systems have been implemented internationally for surveillance, research, and practical improvements [4,5]. Moreover, systems provided feedback on QI [6–9]. Sweden has no national database containing primary care data at the individual level, largely because of integrity concerns. In the PCQ, patient identity is retained at the PCCs, while aggregated data are collected regionally, avoiding the integrity risks associated with large databases. Moreover, the current national visualisation platform ‘Health Care in Numbers’ uses only aggregated data and displays a limited number of PCQ indicators from selected Swedish regions [17]. The new national database for PCQ, which is currently underway, will collect data at an aggregated level and will, in the near future, have the possibility of visualising all PCQ indicators from all Swedish regions. Being the sole source of national clinical data covering a wide range of disease conditions across the entirety of primary care, the national database för PCQ will be a valuable resource for research purposes.

Statistics with aggregated data from the national PCQ database can be used for regional and unit comparisons, time-dependent analyses, and evaluating measures from other data sources like quality registries and government reports. They can also serve as a basis for requesting individual-based data from regional data warehouses to evaluate quality improvement interventions, thus overcoming the limitation of only offering aggregated data.

The PCQ system was developed for audits and feedback on QI [1]. On daily clinical work and possible improvements, data does not require the same accuracy as data for accountability or research [46]. Considerable variability exists in data extraction processes among the 21 Swedish regions, in addition to variability in organisational structures, legal assessments and reimbursement programs and care practices [18]. There are also differences between regions in terms of where data can be retrieved for comparison, such as whether data from both private and public sectors can be accessed from a consolidated regional source, or whether inpatient care and specialist outpatient care treatments can be included, or if medications can be retrieved solely from primary care or also from other specialists outpatient and inpatient care. Thus, the data are usable for local and regional purposes but not yet for national comparisons. Similar challenges in other countries have been described [47]. This variability is one reason for the lack of linking financial incentives to performance based on the indicators in the PCQ. Moreover, international organisations such as EQuiP [48] and WONCA [49] urge refraining from linking financial incentives closely to quality metrics because of the risk of undermining care quality [50]. A few regions in Sweden have linked financial bonuses to QI efforts rather than achieving certain results on quality indicators [51]. By focusing on feedback mechanisms rather than outcome-based financial rewards, PCQ encourages regions to adopt a more sustainable approach to QI, prioritising QI efforts and long-term outcomes over short-term gains.

## Conclusion

The PCQ system is a national framework with an impact on national standardization for indicators in primary care, designed for automatic and systematic data collection and feedback from primary care electronic medical records. PCQ data are available for over 97% of all PCCs in Sweden.

The PCQ aims for QI, consists of indicators developed by professionals, and reflects the width of general practice. The results of quality improvement (QI) efforts, both regionally and locally, can be demonstrated through the use of the PCQ. Data were visualised in real time as graphs, with the possibility of benchmarking at an aggregated level and identifying individuals locally at the PCC. However, there are challenges concerning the variability between regions in the data extraction process and the lack of patient-related outcomes. Moreover, the incentives to put aside time for QI vary between regions.

## Supplementary Material

APPENDIX A_ME.docx
