# Supplementary material for: Enhancing primary care quality improvement through national data collection and validation: the primary care quality initiative in Sweden
Source: Scand J Prim Health Care. 2025 Apr 20;43(3):649–59. doi: 10.1080/02813432.2025.2490921 (PMC12377125; doi:10.1080/02813432.2025.2490921)
Supplement: APPENDIX A_ME.docx [file IPRI_A_2490921_SM1996.docx]

## **APPENDIX A**

**General indicators for primary care**

| **Continuity**  Continuity index (Bice Boxerman) for:   - all patients, last 18 months, for GPs/ nurses - patients with chronic illness, last 18 months/last 3 years, for occupational therapists/ physiotherapists/ GPs/ nurses / psychotherapists - patients with comorbidities 2-3 chronic diseases/ ≥4 chronic diseases - patients with Coordinated Individual Plan GPs/ nurses - patients with leg ulcers, last 18 months, assistant nurse or nurse - patients who are “frequent attenders”   Proportion of patients at the PCC who   - has a with a appointed doctor as “their GP” - had >65% of their doctor contacts with their appointed doctor |
| --- |
| **Lifestyle habits**  Proportion with chronic disease who   - has a registered note on lifestyle habits - has a registered note on alcohol habits/ diet/ physical activity/ tobacco use/   Proportion of daily smokers who received counselling of patients with   - any chronic disease/ - dementia/ depression/ diabetes / heart failure / hypertension /Chronic Obstructive Pulmonary Disease (COPD)/ coronary artery disease /osteoporosis /psoriasis /rheumatoid arthritis /schizophrenia /TIA or stroke   Proportion of patients with insufficient physical activity who received counselling of patients with:   - any chronic disease/ - osteoarthritis/ dementia/ depression/ - diabetes / hypertension /Chronic Obstructive Pulmonary Disease (COPD)/ stress reaction   Proportion of patients with risky alcohol consumption who received counselling of patients with:   - any chronic disease/ - depression/ atrial fibrillation/ hypertension / sleep disorders/ anxiety   Proportion of unhealthy diet habits who received counselling of patients with;   - any chronic disease/ - hypertension |
| **Medication**  Proportion of patients with   - proton pump inhibitors (PPI) who have a registered evidence-based indication - increased cardiovascular risk treated with NSAIDs - benzodiazepines/ benzodiazepines and high consumption/ new treatment who got the smallest package - opioids / excluding patients with cancer diagnoses / new treatment who got the smallest package - NOACs out of all with oral anticoagulant treatment - oral anticoagulants who were followed up with blood tests - alcohol problems treated with relapse prevention - a risky drug combination potassium together with potassium-sparing diuretics/ oral anticoagulants together with ASA/ non-selective calcium antagonists (diltiazem or verapamil) together with beta-blockers - p.o. Diclofenac of all prescribed p.o. NSAIDs - topical Diclofenac out of all who were prescribed topical NSAIDs |
| **Prioritization**  Proportion of patients who have had   - follow-up contact for chronic illness, physically or remotely/ only physically   Proportion of patients with an increased risk of co-morbidity who   - had an exam to estimate risk for cardiovascular disease   Proportion of patients with comorbidity with a a new episode of depression who:   - has drug treatment - psychotherapy   Proportion of patients with chronic disease who   - a team-based rehabilitation plan is recommended for - has a rehabilitation plan / follow up of the plan   Proportion of patients who are “frequent attenders” who:   - are 80 years and older - had many visits to a doctor/ nurse/ occupational therapist/ physiotherapist - has a rehabilitation plan / follow up of the plan |
| **Comorbidity**  Proportion of patients with   - 1 chronic disease (no comorbidity)/ 2 chronic diseases /3 chronic diseases /4 chronic diseases/ 5 or more chronic diseases - Proportion of patients with comorbidity who did not have a doctor's appointment/ had 1-2 doctor's appointments /3-5 doctor's appointments / 6 or more doctor's appointments |
| **Cooperation**  Proportion of patients with chronic disease who:   - had their care needs assessed in primary care or in hospital/ in primary care/ in hospital / neither in primary care or in hospital   Proportion of patients with Chronic Obstructive Pulmonary Disease (COPD)/ TIA or stroke/  heart failure/ coronary artery disease who:   - had their care needs assessed in primary care or in hospital/ in primary care/ in hospital / neither in primary care or in hospital   Proportion of people with a Coordinated Individual Plan (SIP) who had a follow -up contact |
| **Sick leave**  Proportion of patients at the PCC who:   - had a sick leave - had a preventive sick leave   Proportion of patients who had a sick leave   - for depression/ anxiety/ a diagnosis from the musculoskeletal system who received sick leave /exhaustion disorder* |
| **Elderly**  Proportion of patients ≥75 years who   - has medication that should be avoided - has drugs that affect kidney function who had a blood test for kidney function - has oxazepam - has sleeping pills - are at risk of malnutrition |

**Chronic diseases**

| **Osteoarthritis**  Proportion of patients at the PCC who:   - are diagnosed with osteoarthritis   Proportion of patients with osteoarthritis who:   - has a registered weight or BMI   Proportion of patients who has a **new** diagnosis of osteoarthritis and who:   - participated in patient education - had physical training supervised by a physiotherapist |
| --- |
| **Asthma**  Proportion of patients at the PCC who:   - are diagnosed with asthma - has asthma and has regular medication and who had a follow-up visit with any professional/ a GP/ a nurse - has a **new** diagnosis of asthma who did a spirometry |
| **Leg ulcers**  Proportion of patients at the PCC who:   - are diagnosed with leg ulcer   Proportion of patients with a leg ulcer who:   - has an etiological diagnosis - has compression therapy - has an assessment of peripheral circulation registered - had antibiotic treatment |
| **Dementia**  Proportion of patients at the PCC who:   - are diagnosed with dementia   Proportion of patients with dementia who:   - are treated with antipsychotics - had a follow-up visit with any professional/ a GP/ a nurse - has co-morbidity - has a registered weight or BMI - are at risk of malnutrition   Proportion of patients with a **new** diagnosis of dementia   - who have been assessed by an occupational therapist as part of diagnosing dementia - who received counseling about cognitive aids   Proportion of patients with a new diagnosis of Alzheimer's dementia who has tried drug therapy |
| **Diabetes**  Proportion of patients at the PCC who:   - are diagnosed with diabetes type 2   Proportion of patients with type 2 diabetes who:   - has HbA1c <52 / between 52 and 70/ >70 / is without a current value for HbA1c - has blood pressure ≤140/85 mmHg / between 141-149 / 86-89 / ≥150/90 / are without a current value for blood pressure - is treated with statins - had a follow-up visit with any professional/ a GP/ a nurse - not have albuminuria/ has microalbuminuria/ macroalbuminuria / no information on albuminuria - is treated with metformin and who have severely reduced renal function (GFR < 30) - had a foot examination |
| **Atrial fibrillation**  Proportion of patients at the PCC who:   - is diagnosed with atrial fibrillation   Proportion of patients with atrial fibrillation who:   - had a follow-up visit with any professional/ a GP/ a nurse   Proportion of patients with atrial fibrillation who are treated with anticoagulants among those with:   - high risk of stroke - moderately increased risk of stroke - low risk of stroke |
| **Heart failure**  Proportion of patients at the PCC who:   - are diagnosed with heart failure   Proportion of patients with heart failure who:   - had a follow-up visit with any professional/ a GP/ a nurse - has blood pressure ≤140/85 mmHg / between 141-149 / 86-89 / ≥150/90 / are without a current value for blood pressure - had function tests in connection with physical training supervised by a physiotherapist - participated in patient education - has HFrEF who are treated with at least 3 of 4 recommended drug classes   Proportion of patients with a **new** diagnosis of heart failure who:   - had physical training supervised by a physiotherapist |
| **Hypertension**  Proportion of patients at the PCC who:   - are diagnosed with hypertension   Proportion of patients with hypertension who:   - has blood pressure ≤140/90 mmHg / between 141-159 / 91-99/ ≥ 160/100/ are without a current value for blood pressure |
| **Incontinence**  Proportion of patients at the PCC who:   - are diagnosed with urinary incontinence   Proportion of patients with urinary incontinence who:   - had an examination/testing to find out the type of urinary incontinence - had pelvic floor muscle training supervised by a physiotherapist or nurse |
| **COPD**  Proportion of patients at the PCC who:   - are diagnosed with COPD   Proportion of patients with COPD who:   - had an exacerbation and who had a follow-up visit within 6 weeks/ after 6 weeks - has documentation of tobacco use   Proportion of patients with COPD and regular medication who:   - had a follow-up visit with any professional/ a GP/ a nurse - has a registered weight or BMI - are at risk of malnutrition - did a 6-minute walk test - had instructions for energy-saving techniques - had physical training supervised by a physiotherapist - are smokers and who did a spirometry   Proportion of patients with a new diagnosis of COPD who   - participated in patient education - did a spirometry |
| **Coronary artery disease**  Proportion of patients at the PCC who:   - are diagnosed with coronary artery disease   Proportion of patients with coronary artery disease who:   - had a follow-up visit with any professional/ a GP/ a nurse - has statins - has platelet inhibitors and/or anticoagulants - has RAAS inhibitors - has blood pressure ≤140/90 mmHg / between 141-159 / 91-99/ ≥ 160/100/ are without a current value for blood pressure |
| **Chronic kidney disease**  Proportion of patients at the PCC who:   - are diagnosed with chronic kidney disease   Proportion of patients under 80 years of age who have reduced kidney function with GFR < 60 who:   - has a diagnosis of chronic kidney disease - has been checked for albuminuria - has blood pressure ≤ 130/80 - also have macroalbuminuria and who has ACEh/ARB medication - has statins   Proportion of patients under 80 years of age who   - has reduced kidney function with GFR < 30 who have a diagnosis of chronic kidney disease |
| **Osteoporosis**  Proportion of patients at the PCC who:   - are diagnosed with osteoporosis   Proportion of patients 50 years of age or older who:   - has a high risk of osteoporotic fracture   Proportion of patients 50 years of age or older with a new high risk of osteoporotic fracture who:   - has drug treatment against osteoporosis - had physical training / fall prevention training/ balance training supervised by a a physiotherapist   Proportion of patients with bisphosphonate treatment who   - have long-term treatment   Proportion of patients who are treated with drugs that inhibit bone resorption who:   - does not have a current value of kidney function or calcium |
| **Long-term non-malignant pain**  Proportion of patients at the PCC who:   - are diagnosed with long-term non-malignant pain   Proportion of patients with long-term non-malignant pain who:   - received a rehabilitation plan at VC - participated in patient education |
| **TIA and Stroke**  Proportion of patients at the PCC who:   - are diagnosed with TIA or Stroke - are diagnosed with Stroke   Proportion of patients with TIA or ischemic stroke who:   - has statins   Proportion of patients with TIA, ischemic stroke or intracerebral hemorrhage who:   - had a follow-up visit with any professional/ a GP/ a nurse - has blood pressure ≤140/90 mmHg / between 141-159 / 91-99/ ≥ 160/100/ are without a current value for blood pressure   Proportion of patients who had a stroke who   - did supervised ADL-traing - had balance training supervised by a physiotherapist - had physical training with a focus on walking ability supervised by a physiotherapist |

**Mental health**

| **Depression**  Proportion of patients at the PCC who:   - are diagnosed with depression   Proportion of patients with a new episode of depression who:   - had a follow-up contact within 6 weeks - had a follow-up contact and a self-assessment within 6 weeks - had new treatment with antidepressants and who had a follow-up visit within 6-18 months with a GP or a psychologist / any professional - had a physical examination - has antidepressants - received psychological treatment with Cognitive Behavioral Therapy (CBT) - received Interpersonal Psychotherapy (IPT) - received Psychodynamic Therapy (PDT) - received Internet-based cognitive behavioral therapy (iCBT) - received psychoeducational treatment - received any type of psychotherapy - had physical training or guided training or basic body awareness therapy supervised by a physiotherapist - has improved self-rating of depressive symptoms within 6 weeks   Proportion of patients with SSRIs who   - have a registered evidence-based indication |
| --- |
| **Anxiety**  Proportion of patients at the PCC who:   - are diagnosed with anxiety   Proportion of patients with a new diagnosis of anxiety who:   - had a follow-up contact within 6 weeks - had new treatment with antidepressants and who had a follow-up visit within 6-18 months with a GP or a psychologist / any professional - had a physical examination - has antidepressants - received psychological treatment with Cognitive Behavioral Therapy (CBT) - received Internet-based cognitive behavioural therapy (iCBT) - received psychoeducational treatment - received any type of psychotherapy |
| **Stress-related disorders**  Proportion of patients at the PCC who:   - are diagnosed with stress-related disorders - are diagnosed with exhaustion disorder* |

**Infections**

| Diagnoses:   - Common cold, acute sinusitis, pharyngotonsillitis, acute otitis media, pneumonia, acute bronchitis - Acute lower urinary tract infections (separately men and women) - Borrelia, erysipelas, atheroma and abscesses, impetigo, nonspecific skin infection, paronychia   For each of the diagnoses  Number   - of episodes of infectious diagnosis per 1000 listed patients and per 1000 patients contacting the PCC   Proportion of episodes   - prescribed antibiotic (acute urinary tract infection in men, borrelia, erysipelas excluded) - prescribed first-line antibiotic (common cold, acute bronchitis, atheroma and abscesses, paronychia excluded) - with a physical visit (acute urinary tract infection in women and borrelia excluded)   Proportion of positive rapid antigen detection tests for Group A Streptococci (Strep A test) of all Strep A tests  Proportion of episodes with pharyngotonsillitis prescribed antibiotic   - with a positive Strep A test - with a negative Strep A test - without a Strep A test   Proportion of upper respiratory infection (common cold, acute sinusitis, pharyngotonsillitis, acute otitis media) with C-reactive protein test  Proportion of lower respiratory infection (pneumonia and acute bronchitis) with C-reactive protein test  Proportion of antibiotic prescriptions without diagnosis of any common infection |
| --- |

* Lindsater E, Svardman F, Wallert J, Ivanova E, Soderholm A, Fondberg R, et al. Exhaustion disorder: scoping review of research on a recently introduced stress-related diagnosis. BJPsych Open. 2022;8(5):e159.
